# Multidisciplinary approach to genomics research in Africa: the AfriCRAN model

**DOI:** 10.11604/pamj.2015.21.229.7380

**Published:** 2015-07-30

**Authors:** Azeez Butali, Peter Mossey, Nikki Tiffin, Wasiu Adeyemo, Mekonen Eshete, Chrispinanus Mumena, Rosemary Audu, Chika Onwuamah, Pius Agbenorku, Mobolanle Ogunlewe, Adetokunbo Adebola, Hecto Olasoji, Babatunde Aregbesola, Ramat Braimah, Abimibola Oladugba, Ifeanyichukwu Onah, Ezekiel Adebiyi, Peter Olaitan, Lukman Abdur-Rahman, Adebowale Adeyemo

**Affiliations:** 1 Department of Oral Pathology, Radiology and Medicine, College of Dentistry, University of Iowa, Iowa City, IA. U.S.A; 2 Department of Orthodontics, University of Dundee, Scotland. UK; 3 South African National Bioinformatics Institute, University of the Western Cape, Private Bag, X17, Bellville 7535, South Africa; 4 Department of Oral and Maxillofacial Surgery, College of Medicine, University of Lagos, Lagos. Nigeria; 5 Department of surgery School of Medicine Faculty of health sciences Addis Ababa University, Addis Ababa. Ethiopia; 6 Department of Oral and Maxillofacial Surgery, Kigali Health Institute, P.O. Box 3286, Kigali, Rwanda; 7 Human Virology Laboratory, Nigerian Institute of Medical Research, 6, Edmond Crescent, P.M.B. 2013, Yaba, Lagos, Nigeria; 8 Department of Plastic Surgery, Kwame Nkrumah University of Science and Technology, Kumasi, Ghana, P.O. Box 448, KNUST, Kumasi, Ghana; 9 Department of Oral and Maxillofacial Surgery, Aminu Kano University Teaching Hospital, Kano. Nigeria; 10 Department of Oral and Maxillofacial Surgery, University of Maiduguri Teaching Hospital, Maiduguri. Nigeria; 11 Department of Oral and Maxillofacial Surgery, Obafemi Awolowo University, Ile-Ife. Nigeria; 12 Department of Biostatistics, University of Nigeria, Nssukka. Nigeria; 13 Department of Plastic Surgery, National Orthopedic hospital, Enugu. Nigeria; 14 Department of Computer and Information Sciences and Covenant University Bioinformatics Research (CUBRe), Covenant University, Ota, Nigeria; 15 Department of Plastic and Reconstructive Surgery, Ladoke Akintola University Ogbomosho. Nigeria; 16 Department of Pediatrics, University of Ilorin. Nigeria; 17 Center for Research on Genomics and Global Health, National Human Genome Research Institute, National Institutes of Health, Bethesda, MD, U.S.A

**Keywords:** Genomics, Africa, Multidisciplinary team

## Abstract

This article is an outcome of the African Craniofacial Anomalies Research Network (AfriCRAN) Human Hereditary and Health (H3A) grant planning meeting in 2012 in Lagos, Nigeria. It describes the strengths of a multidisciplinary team approach to solving complex genetic traits in the craniofacial region. It also highlights the different components and argues for the composition of similar teams to fast track the discovery of disease genes, diagnostic tools, improved clinical treatment and ultimately prevention of diseases.

## Introduction

As we gradually and deliberately move towards the era of personalized medicine, it is important to understand the diversity that exists in the human genome. Emerging technologies and tools have made it possible to understand the molecular mechanisms underlying disease progression. Scientists are now able to interrogate the genome in order to determine the role of functional loci in coding and non-coding regions [1]. Clearly, we have made tremendous progress in the quest to use the knowledge of the genome to treat and prevent human diseases. Genomic studies in Africa is witnessing the best of times with the establishment of the Human, Hereditary and Health Africa (H3A) initiative supported by the NIH and Welcome Trust. The H3A was established to develop and support a continent-wide network of scientists and laboratories that will use “state of the art” approaches and technologies to the study of the complex interaction between environmental and genetic factors in disease etiology and pathogenicity. This initiative will also explore drug responses in African populations. The ultimate goal of the H3A is to use data obtained from research efforts to influence and inform strategies that will address health inequities. This is line with the vision of the WHO Global Burden of Diseases and the Global Oral Health Inequalities Research Network (GOHIRN) of the International Association for Dental Research. These global efforts and strategies will bring health benefits to Africans and indeed the World. Craniofacial conditions are amongst the most common health disparities and one of the leading causes of health inequality affecting low-income countries in the world [2]. In Africa, craniofacial disparity is being addressed by studying orofacial clefts through the African Craniofacial Anomalies Research Network (AfriCRAN). The African Craniofacial Anomalies Network (AfriCRAN) is a collection of craniofacial researchers in Africa working with international collaborators in order to collect data on genetic variations and environmental exposures from individuals and families with craniofacial abnormalities in Africa. These resources will be used to investigate the etiology of these complex traits and to serve as a basis for more extensive studies in the future. The Network′s major strength is its people, who bring their commitment, and expertise to this collaboration and who will be responsible for the Network's strong continental and international capacity. Orofacial cleft (OFC) is the most common birth defect of the head and one of the most common birth defects in humans. The overall prevalence is 1/700 live births and this differs across ethnic groups [2]. Orfacial cleft serve as a sentinel for all birth defects and therefore a good model for studies on etiology, treatment and prevention. The prevalence of overt OFC in Africa is 0.5/1000 and this is low compared to other populations [3]. Understanding the etiology of OFC in this unique population will provide additional insights into the etiology and this will be important for studies on prevention. In this article, we will highlight how AfriCRAN is leveraging on its multidisciplinary composition to carryout genomic studies on craniofacial anomalies beginning with orofacial clefts.

## Letter to PAMJ editors

### Phenotyping

An essential pre-requisite for studies on human genetics is accurate and reliable phenotyping. Phenotypes are sets of observable characteristics and they are the product of the interaction between genotype (s) and the environment. In other words, phenotypes are the reflections of the nature and the nurture of an organism [4]. Phenotypes can be physical reflections or metabolic profiles that can be measured by some form of validated metrics. In AfriCRAN, our study coordinators in participating country are surgeons in different specialties (plastic surgery, ear nose and throat surgeons, pediatric surgeons, maxillofacial surgeons and dental surgeons). Using their individual and collective expertise, the surgeons and clinicians ensure that eligible cases are accurately phenotyped. For all the cases recruited, the surgeons carry out standardized physical examinations; take clinical photographs and record the full description of cleft phenotypes and all other recognizable malformations. All data obtained are entered into a secured Redcap database [5]. Centers also have access to echocardiogram and electrocardiogram results to rule out other heart defects. A second tier of phenotyping is done in collaboration with international collaborators in Iowa where there is expertise for detecting syndromes. We carry out regular reviews of cases as quality assurance before the genomic investigations. In a recent study, we used molecular techniques to tease out syndromic cases previously classified as non-syndromic clefts [6]. Our strategy is to make every opportunity for surgery available for research. Individuals are recruited through the hospitals and community surgical outreach programs in Nigeria, Ethiopia, Ghana, Kenya and Rwanda. Samples from the affected individual are collected in the hospital by the surgeons who also describe the phenotypes. AfriCRAN enjoys the services of specialist registrars and nurses who are employed as research assistants. These research personnel help in data collection from cleft subjects and their parents as well as unaffected controls.

### Molecular genetics applications

The ultimate goal in any genetic research is to reduce the risk for the disease and to facilitate strategies for prevention. As we approach the era of personalized medicine, studies have argued that it is important to investigate diverse populations in the study of complex diseases [7–9]. The discoveries of candidate genes for orofacial clefts has benefitted from studies that included individuals from different populations. The first gene, IRF6 reported to be significantly associated with Van der Woude syndrome (VWS), Popliteal pterygium syndrome and non-syndromic clefts was first identified in monozygotic twins with Van der Woude syndrome (VWS) from Brazil [10]. Genome-wide association studies (GWAS) provided additional evidence supporting the need to investigate diverse population groups for a complex trait such as CL(P) [11–13]. The African populations serve as the ancestral population to humans around the world and the African genome has accumulated the greatest genetic variations [14, 15]. The genomic era is witnessing the large scale sequencing of many personal genomes to understand disease etiology, mechanisms and diagnosis. This is possible through the success of GWAS for common, complex diseases and the reducing cost of generating data using high throughput technology. A vast majority of GWAS studies including those on non-syndromic orofacial clefts has been conducted in populations of European origin with only a few focused on Asian or African populations. We believe that an investigation of the genetic variations in these related populations across the world affords better opportunities to identify new risk genes/ loci. It will also provide an opportunity to understand the differential contributions of known candidate genes. These opportunities will expand our current understanding of these complex traits and provide the potentials for translating significant findings to affected individuals and families in other populations. These findings will bring forth several opportunities geared towards the design of accurate diagnostic and predictive markers, improved molecularly based treatment and better health for Africans and the world. The AfriCRAN is well suited to carryout genome-wide studies using a large cohort of orofacial cleft samples collected in countries from sub-Saharan Africa. The network works in collaboration with international partners at centers of excellence in Iowa, NIH and Pittsburgh in the United States, Dundee in the United Kingdom and Ottawa in Canada.

### Bio-informatics

We work with scientists at the South African National Bio-informatics Institute (SANBI) who has substantial experience in bioinformatics research and genomics. This group specializes in elucidating the genetics underlying human disease using computational approaches, particularly in the African context. SANBI has the capacity to host a centralized relational database, with appropriate data fidelity, security and backup. Patient and sample details will be entered and accessed by consortium members through a browser-based, user-friendly front-end form. Additionally, SANBI will undertake processing of next generation sequencing data (genome and exome), functional SNP identification, and integration of data generated with gene and pathway analysis to determine clinical and biological meaning of disease-associated variation. Presently, SANBI works closely with the Bioinformatics department at Covenant University, Ogun State, Nigeria, as well as the H3 Africa Bioinformatics Network. SANBI is well-placed for this role, given that development of pipelines for analysis of next generation sequencing data is already underway at the Institute through an NIH funded H3A Bioinformatics Network.

### Bioethics

Craniofacial anomalies are surrounded with considerable superstitions stemming from cultural beliefs in many countries on the African continent. Typically, children born with craniofacial anomalies are associated with ill omens, witchcrafts and are thought to be the consequence of an abuse by the gods on their mothers during pregnancy. The birth of children with orofacial clefts comes with some major concerns that include altercation in the families and infanticide (mainly due to deliberate aspiration of the affected child during breast feeding). Orofacial clefts are a matter of life and death in most part of Africa [16, 17]. Stigmatization of individuals and families is very common in Africa, where there is limited knowledge about the etiology and management of clefts (Adeyemo et al., unpublished) [18]. Across the continent, it is still a challenge to explain the etiology of these phenotypes to families. Translating significant findings from genetic studies that may directly impact on personalized medicine will definitely pose additional challenges. Genomics research into any type of diseases or conditions raise a number of important ethical issues relating to informed consent [19]; privacy and confidentiality [20, 21]; data sharing and secondary use (Foster and Sharp, 2006) [22]. These challenges are very important when research is conducted in countries with low socio-economic indices and education levels [23]. There are also challenges associated with obtaining a valid informed consent [24, 25]. Genetic studies are extremely important and have provided significant insights into the etiology as well as clues to management and prevention [26–28]. They can also reveal hidden secrets about families e.g. risks for cancer, clefts etc [29–31]. Poorly managed information on these risks can add to pre-existing stigmatization, thus posing a challenge to the science community. For instance, in a study examining podoconiosis in Ethiopia, Tekola et al (2009) [25] found that the association of a stigmatized condition with “blood”, descent and family was thought to increase stigma for the entire family, and not just those suffering from the condition (Tekola et al.,2009) [25]. In another study in the US, Phelan et al. (2005) [32] found that even though respondents were less likely to attribute blame for having schizophrenia to patients when a genetic cause was known, they were also less likely to think that the person could improve with treatment (Phelan et al.,2005) [32]. In recognition of socially important issues that can directly affect the success of future genetic studies, AfriCRAN is working with bioethicists across the continent to develop studies that will influence policy changes aimed at reducing and abolishing stigmatization.

### Prospects of a multidisciplinary team

There is improvement in the knowledge of the genome and successes in the application of genomic studies to improve diagnosis and treatment. Multidisciplinary approach is the silver lining in the dark cloud that has befallen genetic studies of complex traits. As more experts avail their skills, techniques and thoughts to teams; it will only be a matter of time for the genomic world to have direct bench to bed side applications.
